# Effects of a blended classroom-based intervention on aerobic fitness, motor skills, inhibition, and daytime sleepiness among Hong Kong children

**DOI:** 10.3389/fpubh.2022.944423

**Published:** 2022-09-20

**Authors:** Ming Hui Li, Cindy Hui Ping Sit, Stephen Heung Sang Wong, Yun Kwok Wing, Ching Kong Ng, James Rudd, Jia Yi Chow, Raymond Kim Wai Sum

**Affiliations:** ^1^Department of Sports Science and Physical Education, The Chinese University of Hong Kong, Shatin, Hong Kong SAR, China; ^2^Department of Psychiatry, Faculty of Medicine, The Chinese University of Hong Kong, Shatin, Hong Kong SAR, China; ^3^Stewards Pooi Kei Primary School, Hong Kong, Hong Kong SAR, China; ^4^Department of Teacher Education and Outdoor Studies, Norwegian School of Sport Sciences, Oslo, Norway; ^5^Physical Education and Sports Science, National Institute of Education, Nanyang Technological University, Singapore, Singapore

**Keywords:** physical literacy, sit-stand desk, children, primary school, motor skill, sleep, inhibition

## Abstract

In response to the all-round development of primary school children, this study aimed to evaluate the effectiveness of a blended intervention program on children's health-related outcomes of aerobic fitness, motor skills, inhibition and daytime sleepiness in classroom settings. Three experimental conditions include: (1) the “Stand + Move” group combining sit-stand desks and physical activity (PA) recess, (2) “Move” group with PA recess only, and (3) Control group (CG; normal class schedule). A total of 64 primary school children (37.5% girls and 62.5% boys, M [SD] = 9.6 [0.61], BMI mean = 17.0 ± 3.0) participated in all assessments, including aerobic fitness, motor skills, inhibitory control, and daytime sleepiness. The baseline data collection starts from January 2019, with the intervention lasting for 13 weeks and followed by post-intervention and follow up tests conducted in July and October 2019. There was a significant interaction effect on aerobic fitness [*F*_(2,76)_ = 10.62, *p* < 0.001, η^2^ = 0.22] after the intervention period, whereas no significant interaction was observed for other variables. Significant main effects were observed in aerobic fitness (pre – post: −11.75 and −7.22) for both experimental groups, with the blended group showing greater improvements immediately post the test, while motor skills only showed a significant increase at the three-month follow-up, with the greatest increase in the blended group (pre-follow-up: −2.50). For inhibition control and daytime sleepiness, better improvements were shown for the experimental groups than for the control group. The blended designed intervention, by incorporating multiple components as an innovative strategy to reconstruct children's traditional classroom environment in Hong Kong, has demonstrated improved physical and psychological development of school children.

**Clinical trial registration:**

ChiCTR, ChiCTR2000035038. Registered 29 July 2020 – Retrospectively registered. http://www.chictr.org.cn/hvshowproject.aspx?id=46038.

## Introduction

A traditional classroom environment tends to encourage prolonged sitting and a sedentary lifestyle in schoolchildren (1). In some countries, evidence shows that over 63% of class time per school day is considered sedentary (2), and the reported sitting time has been as high as 10 h/day (3). In Hong Kong, children spend up to 32.3% of their waking time sitting, with their prolonged sitting time being approximately 4.9 h/school day (4). Previous studies found that excessive sitting was negatively associated with cardio-metabolic health risk markers, including obesity, metabolic syndrome, high blood pressure and cholesterol in children (5, 6). Moreover, this continues throughout childhood into adolescence and adulthood (7). According to recommendations, schools should facilitate at least 60 min moderate-to-vigorous physical activity (MVPA) for children, through a comprehensive approach incorporating both activities during class, recess, and before-and-after school (8). The movement guidelines for children and youth have especially been emphasized with respect to optimal health by combining physical activity (PA), restricted sedentary behavior, and adequate sleep as three co-developmental behaviors related to a balance of movement behaviors within a 24-hour period (9). The comprehensive approach also emphasized the development of children's physical literacy (8), as this encourages an embodiment encompassing “the motivation, confidence, physical competence, knowledge, and understanding to value and take responsibility for engagement in physical activities for life” (10, 11). Therefore, it is important to promote classroom-based interactions to prevent prolonged sitting and enhance adequate PA as a novel strategy to foster a healthy lifestyle in children throughout their life (12).

A substantial body of evidence from classroom-based interventions shows that sit-stand desk installation is one of the effective interventions for reducing children's prolonged sitting time without affecting their academic achievement (13, 14). Both, a full-desk (a sit-stand desk for every child) and partial desk allocation (limited for children to share and rotate), have provided meaningful contributions to low intensity daily PA (1, 15), yet a full-desk allocation could guarantee optimal health benefits for children as they would have maximum exposure to the desks (16). However, considering financial feasibility, only three studies have adopted the full-desk system (3, 16, 17) and these were usually limited to one school. Regarding the outcomes in the field of children's physical and psychological health, the use of sit-stand desks or standing desks in primary schools has shown mixed effects on energy expenditure, PA, muscular comfort, and concentration, as related to diverse assessments and measurements with low statistical power (18). This systematic review examined the impact of standing desks in 11 studies and showed that such a strategy could be beneficial for some outcomes, including the enhancement of children's reduction in sedentary behavior. However, as a single component intervention design, it has less positive effects when compared with a multicomponent intervention including educational and practical components such as information on health and posture, the creation of a classroom that encourages movement, and a standing workstation area (18). In this case, a multicomponent intervention “package” may be more beneficial for engaging a wider range of needs and interests within classroom settings by integrating PA opportunities and reducing sedentary behavior during school days. Active breaks during recess have been shown to be effective in promoting PA in children and adolescents (19). A recent systematic meta-analysis found a statistically significant trend for PA and step counts, while the outcomes regarding classroom behaviors to those of cognitive functions and academic achievement did not provide conclusive findings in the meta-analysis due to the heterogeneity of the studies (20). McMichan, Gibson (21) also reported that solely PA or sedentary behavior interventions showed a small or even non-significant effect related to limited empirical studies. As such, a blended interventional design that modifies the traditional classroom environment by integrating the implementation of PA and breaking up prolonged sitting seems to be a promising and feasible long-term strategy for enhancing physical and psychological development and fostering physical literacy. Therefore, this study adopts a blended “Stand + Move” design to reduce prolonged sitting and increase school-hour PA engagement through environmental modifications in classroom settings.

Previous research has reported a significant increase in sedentary behavior of children aged 11 years and older, relative to younger age groups (22). Thus, it is necessary to reduce typically observed prolonged sitting among primary school children before transitioning into adolescence. PA recess strategies have also been shown to improve children's motor and cognitive development (23). The physiological and psychological aspects were especially emphasized when evaluating the effectiveness of this blended program while considering targeted outcomes. A previous school-based PA intervention has shown beneficial long-term effects on aerobic fitness performance after a 3 years follow-up (24). An updated review by Kriemler, Meyer (25) found that multicomponent approaches in children during school-based activities have the highest level of evidence for increasing overall PA and fitness, while only 11 of 20 included studies focused on the intervention effects of promoting aerobic fitness. However, motor skills have also been purported as contributing to children's physical, cognitive and social development (26). The study conducted by Ohlinger, Horn (27) investigated the effect of an active workstation on motor skills among university employees while they were in different situations of sitting, standing, or walking. As motor skill development should be a key component in childhood interventions aiming to promote long-term PA (28), it is necessary to include motor skills as one of the outcome variables within the physiological aspect of children's development.

A previous systematic review highlighted the evidence that higher levels of motor skills and cardiorespiratory fitness can contribute to improved cognitive capacity and academic performance in children (29). There were a number of studies evaluating the impact of sit-stand desks on children's executive functions (16, 27, 30), as it contributed to cognitive, academic and overall health-related (physical, metabolic and mental health) outcomes among children and adolescents (31). Additionally, previous studies have also reported that daytime sleepiness is a factor that can impact working productivity of adults (32), while when embedded into real-time settings within school, it would be related to children's effectiveness in learning or even academic performance. All these factors that work together can impact health-related quality of life (33). Having a comprehensive picture guided by the concept of physical literacy, classroom-based interventions to improve health-related outcomes of children and adolescents are required to include various physiological and psychological aspects as outcome variables, including aerobic fitness, motor skills, executive functions, and sleep.

As informed by Sherry, Pearson (18), blended interventional studies targeting sit-stand desks and PA recess not only need to include various health-related outcome variables but also more high-quality study designs. Thus, this study incorporates sit-stand desks and PA recess as novel strategies to benefit the physical and psychological aspects of students in Hong Kong primary schools. It was hypothesized that the blended “Stand + Move” group, compared with the single “Move” and control groups, improved significantly in (1) aerobic fitness, (2) motor skills, (3) inhibitory control, and (4) daytime sleepiness.

## Methods

### Study design

The “Stand + Move” intervention was designed as a three-arm RCT study, which focused on approximately 9-year-old (4th grade of primary school) students from Hong Kong primary schools. One public school from the New Territories, Hong Kong has been approached and invited to participate. The convenience sampling has been adopted to recruit all the grade 4 students in this school. All the participants were randomly assigned (using Google random number generator) to either control or intervention conditions. Outcome data were collected at baseline, post-intervention and 3-month follow-up for all the variables. Baseline data collection was conducted before the intervention began in January 2019, the post-intervention measure for all the participants was performed successively 13-week after the completion of the intervention during the end of semester in July 2019, and the follow-up measurement was performed 3 months after at the start of the next semester (October 2019). Reporting of the trial follows the CONSORT statement. The study received ethics approval from Survey and Behavioral Research Ethics of the university (Reference No. SBRE 18-108) and all the participants provided written parental consent forms prior to participation.

### Participants and intervention

A sample size of 20 in each group [recruiting 24 with an assumed 20% attrition (34)] would have at least power of 80%, an alpha of 0.05, and effect size (*r*) of 0.3 (35) calculated by G-power software. Children were excluded if they had a disability that prevented standing, or had an injury or illness that limited performing normal daily tasks. All the participants were randomly assigned to either of the three conditions with a 1:1:1 ratio, which included: a blended intervention “Stand + Move” group, a single PA break “Move” intervention group, and a control group (CG). All the participants (i.e., students) were blinded to either the group allocation or the research aims, but the intervention providers (i.e., school teachers, research assistants, etc.) could not be blinded.

The height-adjustable sit-stand desks (Askisi 720, SMART Inc., USA) were placed in the “Stand + Move” students' classroom for two academic semesters. Similar to Hinckson, Salmon (1)'s descriptions, the desk could be moved up and down manually with the use of a lever which would allow the children to work in a seated or standing position. Prior to the intervention phase, a three-hour briefing session regarding the instructions on how to administer the sit-stand desks and PA-based recess was held for all the teachers and parents in order to support them in the development of classroom environment within schools. The research plan of breaking up prolonged sitting every 15 min during two regular classes (each class before the recess) per day could ensure all children in the “Stand + Move” group use the sit-stand desks for at least 1 h per day on average across the week (30). Stools or chairs were retained in the classroom for them to feel free to choose whether they sat or stood when using the sit-stand desks. Teachers encouraged each child to stand at the end of 15-min prolonged sitting. Besides, “Stand + Move” and “Move” children participated in a PA recess during recess time, in which the unstructured outdoor interactive games were introduced to children by PE interns. The PA breaks were up to 15 min in duration and twice a day across the week, which included games such as skipping rope, shuttlecock kicking, hide-and-seek in the specific area, and supplemented with several minutes of cooling down. The students who were assigned to the control condition adhered to their regular class schedules and lesson delivery format. They used their standard classroom desks in the classroom, with no experimental changes made to the environment, and there were no changes or specific activity during their recess. Details of the intervention were described elsewhere in a protocol study (15).

### Outcome measures and procedures

Children's height and weight were measured by trained appraisers using standardized procedures, with children in light clothing and shoes removed, using TANITA measuring boards (RD-545-sv) and Seca model 770 scales. Body mass index (BMI, kg/m^2^) was then calculated from the measured weight (nearest 0.1 kg) and height (nearest 0.1 cm), with the standard equation (body weight [kg]/height [m^2^]).

Aerobic fitness was measured using the FitnessGram 15/20 m Progressive Aerobic Cardiovascular Endurance Run (PACER) (36), which was chosen as the stand-alone measure of aerobic fitness according to its strong correlation with maximum oxygen consumption (*r* = 0.83) (37). The PACER consists of a multistage progressive 15- or 20-m shuttle run requiring students to run laps between two markers in time with prerecorded audible beeps. The time between beeps decreases each minute, requiring a progressive increase in pace, and students run laps until they are unable to finish before the beep on two separate occasions. Due to limited space, all participants in this study ran between two markers set 15 m apart, while keeping pace with a prerecorded Cantonese cadence. The PACER determines aerobic fitness status based on the number of laps completed.

Children's motor skills were measured by the Canadian Agility and Movement Skill Assessment (CAMSA) (38), a sequence test combining fundamental, complex and combined movement skills, such as catching, throwing, skipping, and hopping, for assessing motor competence. Two trained appraisers performed the CAMSA demonstrations twice, with the first progressing slowly through the entire course, along with a detailed verbal description of each skill. For the second demonstration, the appraiser moved at full speed, while maintaining skill accuracy. Each participant performed four trials, two practice, two timed tests, with the best score of the two timed tests as their final grade. All the practices and timed tests have followed the same procedures and have been conducted consecutively. All performances were videotaped for verification of skill accuracy by a third appraiser (39). The score of CAMSA test was composed of time score (14 points) and skill score (14 points), resulting in a total of 28 points (38).

Children's executive inhibitory control was examined using a modified version of the Eriksen flanker task (40) with the Inquisit 5 platform. Participants were required to perform the task in a quiet room under the supervision of an instructor who was trained prior to the testing. This task consisted of five arrows on a screen, and participants were asked to determine the direction of the target arrow in the middle. The two flanker arrows on each side of the target arrow worked as the distractors and would appear as either congruent trial “>>>>>” or congruent trial “>><>>.” Each stimulus was shown for 120 milliseconds (ms), and the participants were required to respond within 200 to 1750 ms from the onset of the arrows, for a valid response. This task contained four practice trials and 20 test trials, with an equal number of congruent and incongruent trials occurring in a random order. The main dependent variables were accuracy percentage and mean reaction time (RT).

Daytime sleepiness was assessed using the Pediatric Daytime Sleepiness Scale (PDSS) (41). This was a parent-reported instrument consisting of 8 items, having > 0.40 acceptable factor loadings. Higher scores on PDSS were associated with reduced total sleep time, poorer school achievement, poorer anger control, and frequent illness Internal consistency of the total 8-item scale (factor 1, PDSS) was α = 0.81/0.80 for the split-half samples.

### Statistical analysis

Descriptive statistics were expressed as means, and standard deviations. All data was imported into SPSS version 23 for analysis. An α level of 0.05 was used for all statistical tests. Shapiro–Wilk and Levene tests were used to check the normality and homogeneity of data. Multivariate analysis of variance (MANOVA) test were used to assess between group comparisons at baseline. A two-factor mixed-design ANOVA was conducted to assess the change in dependent variables over the 3 time points between groups, separately. Adjustments were made for sex, age and BMI category. Effect Sizes (ES) using partial eta squared were calculated and reported, large effect with η^2^ ≥ 0.14, medium effect with 0.14 > η^2^ ≥ 0.06, and η^2^ < 0.06 indicating small effects.

## Results

**Figure 1:**
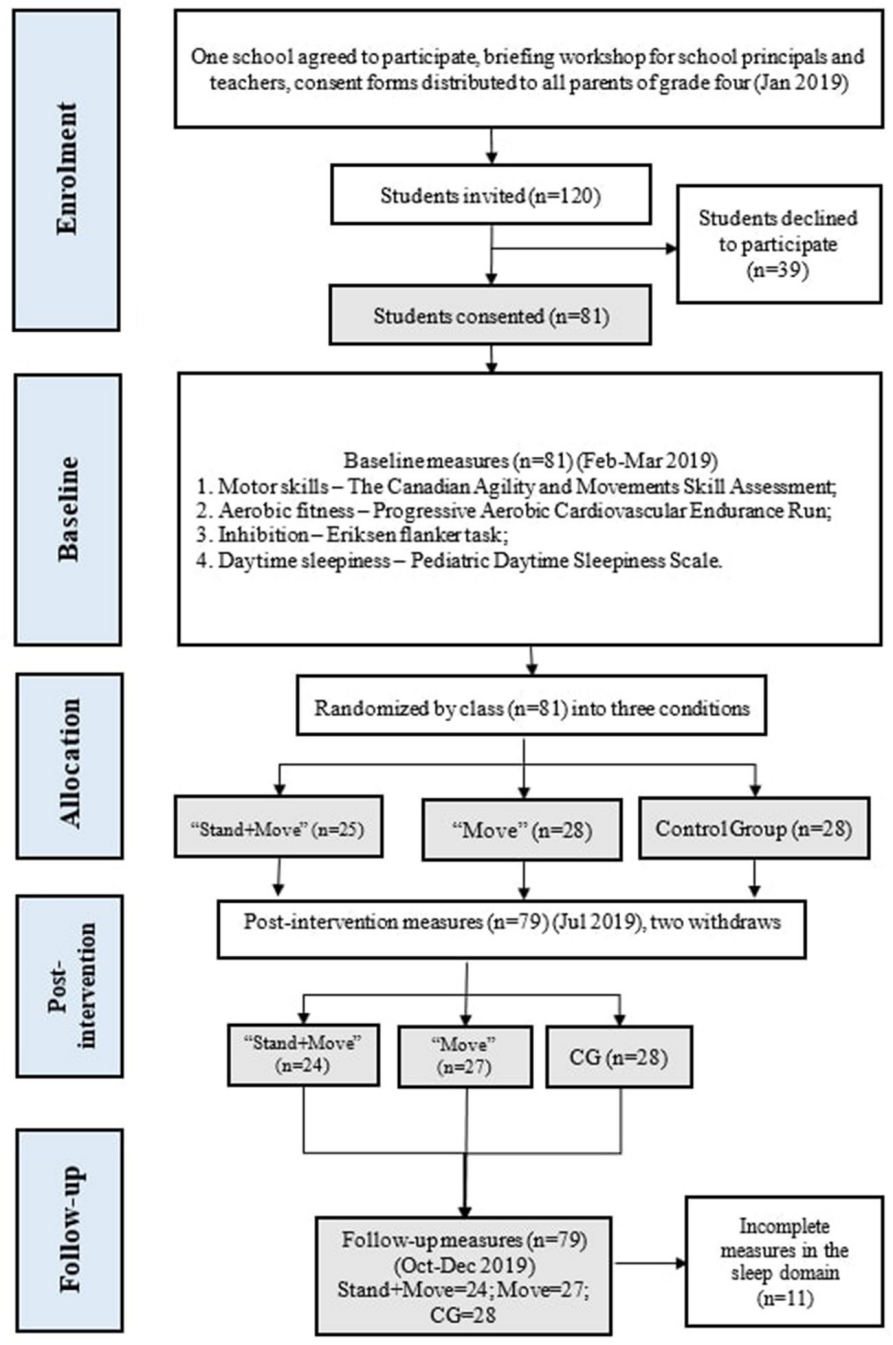
Flow diagram for the blended classroom intervention.

All the grade four students (*n* = 120) were invited and received parent consent forms, and 81 students (response rate = 67.5%) agreed to participate. Of the total sample, 2 dropped out during the intervention, leaving 79 participants (59.5% girls, Mage = 9.6 years [SD = 0.61, range 9.0–12.0]) included for the final analysis. Daytime sleepiness was not available for 11 participants (“Stand + Move” = 3; “Move” = 2; CG = 5) as the questionnaires were not returned from their parents. Figure 1 outlines the flow diagram for the blended intervention. There were no significant differences (*p* < 0.05) between control and intervention groups at baseline for all measured variables. Descriptive statistics of baseline measured variables are presented in Table 1. Intervention effects of the blended “Stand + Move” intervention on all the studied variables over three measurement periods are shown in Table 2. Table 3 displays the mean changes in the measured variables from baseline to immediately post-intervention, while Table 4 displays that from baseline to 3-month follow-up.

**Table 1:** Baseline characteristics of study participants.

	**Stand + Move** **(*n =* 24)**	**Move** **(*n =* 27)**	**CG** **(*n =* 28)**	***p* =**
Age	9.7 ± 0.7	9.6 ± 0.6	9.6 ± 0.6	0.702
Female	15 (60%)	16 (57.1%)	17 (60.7%)	0.645
BMI (kg/m^2^)	16.8 ± 3.0	17.3 ± 3.1	16.9 ± 2.8	0.739
*Aerobic fitness (15 m PACER laps)*	22.9 ± 12.8	24.0 ± 13.0	23.3 ± 11.0	0.981
*Motor skills (CAMSA, 1–24)*	19.9 ± 4.1	19.4 ± 3.7	20.5 ± 2.8	0.548
*Inhibition Control*
Accuracy – congruent (%)	99.6 ± 2.0	97.8 ± 4.2	98.6 ± 4.5	0.355
Accuracy – incongruent (%)	94.2 ± 18.6	90.0 ± 21.3	95.0 ± 12.0	0.580
RT – congruent (ms)	698.8 ± 203.7	642.2 ± 174.1	626.6 ± 167.4	0.256
RT – incongruent (ms)	795.6 ± 353.2	732.7 ± 239.0	774.7 ± 338.6	0.769
*Daytime sleepiness*	13.6 ± 5.6	14.6 ± 5.0	13.5 ± 5.8	0.786

BMI, Body mass index; CAMSA, The Canadian Agility and Movements Skill Assessment; Pacer, Progressive Aerobic Cardiovascular Endurance Run; RT, reaction time.

**Table 2:** Characteristics and interventional interaction effects on the studied variables; Mean ± SD.

**Outcome**	**Post-intervention (*n* = 79)**			Group*Time *P*-value	**Effect size**	**Follow-up (*n* = 79)**			Group*Time *P*-value	**Effect size**
	**Stand + Move**	**Move**	**CG**			**Stand + Move**	**Move**	**CG**		
*Aerobic fitness*	34.6 ± 17.7	31.2 ± 14.3	24.9 ± 12.3	<0.001*	0.22	24.4 ± 11.1	25.9 ± 12.3	24.1 ± 11.8	0.90	0.003
*Motor skills*	19.8 ± 4.6	21.4 ± 3.1	21.3 ± 2.6	0.26	0.04	22.4 ± 2.6	21.4 ± 3.2	22.1 ± 2.3	0.58	0.01
*Inhibitory Control*		
Accuracy-congruent (%)	99.6 ± 2.0	98.2 ± 4.8	97.1 ± 6.0	0.47	0.02	99.6 ± 2.1	96.4 ± 12.2	97.9 ± 5.0	0.82	0.01
Accuracy-incongruent (%)	97.1 ± 4.6	94.4 ± 12.8	95.0 ± 13.2	0.65	0.01	97.4 ± 4.5	92.0 ± 17.1	96.1 ± 5.7	0.88	0.004
RT – congruent (ms)	595.5 ± 154.4	541.4 ± 135.1	583.0 ± 151.3	0.51	0.02	468.8 ± 88.9	472.1 ± 97.3	510.8 ± 112.0	0.85	0.004
RT – incongruent (ms)	599.5 ± 137.0	615.3 ± 163.7	744.5 ± 466.7	0.11	0.06	509.7 ± 94.4	529.4 ± 96.7	518.8 ± 102.1	0.72	0.01
*Daytimesleepiness* ^a^	13.3 ± 5.1	15.6 ± 5.0	13.5 ± 4.7	0.34	0.03	15.1 ± 4.5	16.0 ± 5.4	14.1 ± 4.1	0.28	0.04

^a^n = 68;

* p < 0.05. SD, standard deviation; CAMSA, The Canadian Agility and Movements Skill Assessment; Pacer, Progressive Aerobic Cardiovascular Endurance Run; RT, reaction time.

**Table 3:** Adjusted mean changes (95% CI) from baseline to post-intervention.

	**Stand + Move (*n =* 24)**	**Move (*n =* 27)**	**CG (*n =* 28)**	**ES (partial η^2^)**
*Aerobic fitness*	**−11.75 (−16.57**, **−6.93)**	**−7.22 (−10.88**, **−3.56)**	−1.64 (−5.14, 1.86)	0.185
*Motor skills*	0.17 (−1.22, 1.55)	−1.89 (−5.59, 1.81)	−0.79 (−1.84, 0.27)	0.107
*Inhibitory Control*
Accuracy-congruent (%)	0.00 (−0.01, −0.01)	0.00 (−0.03, 0.03)	0.01 (−0.01, 0.04)	0.013
Accuracy-incongruent (%)	−0.04 (−0.15, 0.08)	−0.04 (−0.16, 0.08)	0.00 (−0.07, 0.07)	0.019
RT – congruent (ms)	**100.8 (14.5, 187.1)**	**97.8 (2.5, 193.1)**	40.3 (−39.2, 119.8)	0.198
RT – incongruent (ms)	**198.1 (37.5, 358.7)**	**117.4 (50.5, 184.4)**	20.4 (−231.0, 271.8)	0.105
*Daytime sleepiness*	**1.47 (0.06, 2.89)**	0.59 (−0.58, 1.76)	2.08 (−0.04, 4.22)	0.195

Bold values signifies p < 0.05. ^*^p < 0.05 for time. CG, control group; ES, effect size; RT, reaction time.

**Table 4:** Adjusted mean changes (95% CI) from baseline to follow-up.

	**Stand + Move (*n =* 24)**	**Move (*n =* 27)**	**CG (*n =* 28)**	**ES (partial η^2^)**
*Aerobic fitness*	−1.50 (−6.89, 3.89)	−1.89 (−5.59, 1.81)	−0.89 (−3.83, 2.04)	0.010
*Motor skill*	**−2.50 (−3.81**, **−1.19)**	**−1.93 (−3.69**, **−0.23)**	**−1.63 (−2.96**, **−0.32)**	0.318
*Inhibitory control*
Accuracy-congruent (%)	0.00 (−0.01, −0.01)	0.02 (-0.05, 0.08)	0.01 (−0.03, 0.04)	0.013
Accuracy-incongruent (%)	−0.04 (−0.14, 0.08)	−0.02 (−0.14, 0.10)	−0.01 (−0.08, 0.06)	0.019
RT – congruent (ms)*	**170.2 (93.5, 246.8)**	**192.3 (114.9, 269.7)**	**148.8 (67.8, 229.8)**	0.493
RT – incongruent (ms)*	**280.1 (88.2, 472.0)**	**255.7 (86.9, 424.5)**	**214.0 (128.0, 300.0)**	0.434
*Daytime sleepiness*	1.90 (−1.07, 4.86)	**2.36 (0.05, 4.68)**	**3.39 (1.27, 5.51)**	0.267

Bold values signifies p < 0.05.

* p < 0.05 for time (whole group). CG, control group; ES, effect size; RT, reaction time.

There were significant Time x Group interaction effect on aerobic fitness [*F*_(2,76)_ = 10.62, *p* < 0.001, η^2^ = 0.22] from baseline to post-intervention, while no significant interaction effect was found for either motor skill [*F*_(2,76)_ = 1.39, *p* = 0.26, η^2^ = 0.04], inhibition RT [*F*_(1,67)_ = 1.41, *p* = 0.25, η^2^ = 0.04], inhibition accuracy [*F*_(2,67)_ = 0.47, *p* = 0.63, η^2^ = 0.01], or daytime sleepiness [*F*_(1,67)_ = 0.07, *p* = 0.79, η^2^ = 0.03] from baseline to post-intervention. During the period from baseline to follow-up, all variables displayed no significant interaction effect (all *P* > 0.05).

For the main effect from baseline to post-intervention, the blended intervention group and “Move” group significantly increased aerobic fitness (pre – post: −11.75 and −7.22), with blended group showing more improvement. While significant reductions were observed in the intervention groups, with the blended group showing less RT in congruent tests (“Stand + Move”: 100.8 ms and “Move”: 97.8 ms) and incongruent tests (“Stand + Move”: 198.1 ms and “Move”: 117.4 ms). At post-intervention, it was interesting to observe that only the blended intervention group showed a significant reduction in daytime sleepiness during this period.

From baseline to follow up, all the groups significantly improved in the measurement of motor skill, with the greatest increase in the blended group (pre-follow up: −2.50). Both reaction time of the congruent and incongruent trials were reduced resulting in better performances in the inhibition tasks performed by two intervention groups and a control group. Participants from “Move” group acquired the largest decrease in the congruent reaction time (192.3 ms [95% CI: 114.9, 269.7]) compared to other two groups, while participants from “Stand + Move” group had the highest mean differences (280.1 ms [95% CI: 788.2, 472.0]) in incongruent responses corresponding to an improved inhibitory control. In addition, there were significant decrease for daytime sleepiness in both “Move” group and CG at follow-up measurements but not for the blended intervention group.

## Discussion

This study represents a randomized controlled trial examining the effects of the blended intervention of combining sit-stand desks and PA breaks as a novel strategy for children's health-related outcomes, including aerobic fitness, motor skill, executive control, and daytime sleepiness. Based on the overall findings, this study showed significantly positive interaction effects in aerobic fitness at post-test and main effects for executive control and daytime sleepiness in the intervention groups. The blended classroom-based research design effectively provides more PA opportunities for primary school-aged children for improving their physical and psychological health-related outcomes. As it is the first classroom-based intervention incorporating sit-stand desks and PA recess as the novel strategy to be evaluated in Asia, the compelling evidence benefits children's physical and psychological health in the longer-term (16, 17).

Overall, the intervention had a large positive influence on children's aerobic fitness (η^2^ = 0.22) from baseline to post-intervention, whereas no time-group interactions were observed from baseline to follow-up. This is highlighted by adopting a blended “Stand + Move” program that might provide opportunities to have an acute effect on children's fitness levels. Incorporating sit-stand desks, together with PA breaks, supplemented with teacher training, can be effective in reducing youth sedentary behavior and increasing PA opportunities in classrooms and recess (42). As such, this study supported that more PA opportunities would help enhance the fitness level, which is consistent with an updated systematic review by Neil-Sztramko, Caldwell (43). They found that school-based PA interventions are effective in increasing maximal oxygen uptake or aerobic capacity, reflecting the physical fitness level of an individual (43). There was no significant interaction effect at the 3-month follow-up, which may be related to the cessation of standing desks after the intervention. A previous short-term PA intervention could be compared with the current study, which also focused on the short-term effect leading to positive changes in the aerobic fitness of children (44). However, their study only targeted children with obesity and did not include a control group for comparison. Therefore, this study might provide insightful evidence and could be generalized to a greater population with better design when incorporating sit-stand desks and PA recess as an interventional strategy (18).

In addition, a statistically significant increase in motor skill competence was perceived at follow-up, albeit with no time-group interactions. The results indicated that the blended intervention strategy might have a longer-term effect on motor skill competence than an immediate effect. This interesting finding was in line with Stodden, Goodway (45) developmental mechanisms of PA trajectories in children, in which the reciprocal relationship toward PA was highlighted with motor skill competence development across the lifespan. It indicated that with the maturing of muscles, bones, and engagement in PA, motor skill competence would be improved during the developmental phase (46). More recently, Barnett, Webster (47) systematically compiled mediation, longitudinal, and experimental evidence to support Stodden et al.'s conceptual model. They found strong positive evidence for the fitness-mediated motor competence/PA pathway in both directions, especially in longitudinal studies across childhood and adolescence. As such, PA interventions might be more effective for motor competence/skills in the long term, considering the developmental pathway between motor skills and PA (47). In addition, the outcome variable was measured through a selected group of fundamental, complex, and combined movement skills within one sequence, with the score including both completion time and skill accuracy scores, rather than individual skills scored by the traditional measurements, that is, Test of Gross Motor Development, Second Edition (TGMD-2) (48). Regarding the multifaceted dimensions and components of CAMSA assessment when scoring motor skills, the final score of this outcome variable may be influenced by multiple components, which might be attributed to the lack of significance for motor skill competence in the short-term post intervention.

Another novel finding of this study was the significant impact of the blended “Stand + Move” intervention on children's executive inhibition control (i.e., reduced reaction time for both congruent and incongruent tasks). This was in line with a meta-analysis that investigated the effects of PA participation on multiple domains of executive functions, which found a positive effect for PA on various subdomains of executive functions (49). Neurocognitive inhibitory control, pertaining to the core executive functions, has been involved in self-controlled behavior, which was under the broader umbrella construct of different disciplines and encompasses cognitive, behavioral, and emotional components, such as inattention, impulsivity, and emotional self-regulation (50). A systematic review showed the overall beneficial effects of PA on executive functions (51), which provided insights into the significance of inhibition in this study. However, heterogeneity of studies and inconsistency of results across studies (52) exists. The inhibitory control can be measured with different tasks. For example, Sjowall, Thorell (53) identified no effects of long-term PA intervention on inhibition control, while their measure used the interference trial from the Color Word Interference Test from D-KEFS (54), which assesses verbal and non-verbal executive functions in children and adults. In this study, the Eriksen flanker task gave the opportunities to disentangle stimulus-level interference from response-level interference (55). The results supported that integrating cognitive and physical demands in a blended PA intervention would be beneficial to enhance motor and cognitive exercise complexity and the affective and motivational factors involved in skill acquisition (56). Such interesting findings and blended research designs have enriched the scope toward the demand for pioneering PA interventions and may provide a theoretical rationale for PA effects on the development of self-control skills (50). It would shed light on applying the theory of ecological dynamics on cognitive performance elicited by PA engagement to inform an individualized enrichment toward the physical literacy journey throughout the lifespan (57).

Regarding the sleep of school-aged children, few studies have examined the effects of a blended “Stand + Move” program that incorporates sit-stand desks and PA recess through a comprehensive physical literacy approach (8), especially considering daytime sleepiness as a result of long-term PA intervention. Previous studies have found that excessive daytime sleepiness affects children's brain functions, including behavioral, cognitive, and health aspects (58), which negatively impacts their school performance and is regarded as a public health concern among children and adolescents (59). A recent systematic review reported that PA programs positively affected various aspects of sleep using both subjective and objective measures, although their population focused on healthy older adults (60). More recently, a cross-sectional study has presented consistent findings that more PA engagement may decrease adolescents' excessive daytime sleepiness, and that daytime sleepiness would increase with a more sedentary lifestyle, especially with prolonged screen time (59). As an emergent field of study, most studies have focused on exploring the associations between PA, sleep, (61–63) and children's sleep behavior to see whether they meet the 24-h Movement Guidelines for Children and Youth (9, 64–66). Few studies have focused on the intervention's effect on daytime sleepiness (67). There is also a need to incorporate specific sleep interventions to enhance the co-development of PA, sedentary behavior, and sleep to enhance children's physical and psychological health with regard to aerobic fitness, motor skills, and cognitive function (68–70). Previous research investigating the relationship between PA and daytime sleepiness may provide evidence that the two domains are significantly associated, suggesting that PA could be one of the factors preventing daytime sleepiness in children aged 9–12 years (67). Considering the harmful effects of excessive daytime sleepiness among children and adolescents (71), the current blended intervention combining PA promotion and strategies to reduce sedentary time may be timely in ameliorating excessive daytime sleepiness in young children.

One of the strengths for this study was the blended design of this study, as it adopted a multicomponent intervention “package” as a novel strategy in school settings (18) and such strategy could provide important evidence to promote children's physical and psychological health to foster a healthy and active lifestyle in children throughout their lifespan (10, 12). As the results shown, a blended research design should be favorably recommended to inform the intervention implementation, combining both quality and quantity of PA included into the “package”, especially considering the need to develop physical literacy as a comprehensive construct within the duration of one academic semester and include different intensities of both structured and unstructured activities, which was regarded as the favorable practical recommendations to inform educational activities. In addition, this study supports Cairney, Dudley (72) evidence-informed theoretical model, with current findings supporting the link between physical literacy, PA, and health. The model positioned physical literacy as a determinant of health, and bidirectional associations existed between the constructs of PA, physical literacy, and health outcomes. This study adopted a comprehensive strategy for PA promotion, combining the reduction of sedentary time, which can lead to a variety of positive physiological, psychological, and social adaptations that benefit the development of physical literacy. Meanwhile, the improved health-related outcomes in physical and psychological aspects could make efforts to improve individuals' PA in various settings. Last, this study may also be valuable in contributing to the physical literacy development of the children of Hong Kong children through a comprehensive approach (8).

Nevertheless, the power of this study was limited by its small sample size. Only one school was recruited for this study, as our intervention content was designed to be cognizant of locational factors, such as cultural norms, the education system, and prevailing teaching styles. Therefore, the present intervention should be interpreted with caution regarding the generalizability of the findings.

## Conclusion

This is the first classroom-based intervention incorporating sit-stand desks and PA recess as a novel strategy to be evaluated in Asia, and only the third study adopting a full-desk allocation system (16, 17). This blended “Stand + Move” intervention led to short-term improvement in aerobic fitness, executive function, and longer-term improvement in motor skills and executive function in school children. The improvement in daytime sleepiness is encouraging; however, future studies should consider incorporating specific sleep interventions into the co-development with PA intervention and the blended novel intervention strategy could adopted for the scaling up research to benefit more primary school children. Overall, this blended classroom-based intervention provides compelling evidence for a cost-friendly and feasible strategy to enhance children's physical and psychological health.

## Data availability statement

The raw data supporting the conclusions of this article will be made available by the authors, without undue reservation.

## Ethics statement

The studies involving human participants were reviewed and approved by Survey and Behavioral Research Ethics. Written informed consent to participate in this study was provided by the participants legal guardian/next of kin. Written informed consent was obtained from the individual(s) and minor(s)' legal guardian/next of kin, for the publication of any potentially identifiable images or data included in this article.

## Author contributions

RS, CS, and ML conceived the study. CS and SW provided guidance and support throughout the study. YW provided professional suggestions in children's sleep measures. CN provide great help for data collection. JR and JC commented the contents, data analysis, and revisions. All authors were involved in the study design, assisted with the drafting, revising of the manuscript, read, and approved the final manuscript.

## Funding

This work was funded by the Direct Grant for Research (Grant No. EDU 2019–052) of the Chinese University of Hong Kong. The funding body did not take part in the design of the study, the collection, analysis, and interpretation of data, and preparation of the manuscript.

## Conflict of interest

The authors declare that the research was conducted in the absence of any commercial or financial relationships that could be construed as a potential conflict of interest.

## Publisher's note

All claims expressed in this article are solely those of the authors and do not necessarily represent those of their affiliated organizations, or those of the publisher, the editors and the reviewers. Any product that may be evaluated in this article, or claim that may be made by its manufacturer, is not guaranteed or endorsed by the publisher.
